# Does your surname undermine your research impact?

**DOI:** 10.3758/s13423-025-02727-0

**Published:** 2025-08-04

**Authors:** Yuejun Lawrance Cai, Kin Fai Ellick Wong, Jessica Y. Y. Kwong

**Affiliations:** 1 Department of Management and Strategy, The University of Hong Kong, Pok Fu Lam, Hong Kong; 2 Department of Management, School of Business and Management, Hong Kong University of Science and Technology, Clear Water Bay, Kowloon, Hong Kong; 3 Department of Marketing, The Chinese University of Hong Kong, Ma Liu Shui, Hong Kong

**Keywords:** Alphabetical order, Citation frequency, Primary effect, Research evaluation

## Abstract

Citation frequency is widely recognized as a crucial metric for assessing academic impact. Previous studies analyzing data from citation databases have observed a surname order bias—a phenomenon where the alphabetical ordering of researchers’ surnames negatively impacts their citation counts. However, the underlying mechanisms driving this bias, the causality behind it, and its implications for in-text citation practices remain poorly understood. Therefore, the present research aims to address these gaps through two preregistered studies. Study 1 replicates and extends the work of Stevens and Duque (*Psychonomic Bulletin & Review*, *26*, 1020–1026, 2019), using a larger sample of 446,755 articles and controlling for surname initial frequency and publication year. Study 2 is an experiment with 307 valid responses from academics holding doctoral degrees, manipulating both citation systems and surname alphabetical order. Consistent and robust findings emerged across both studies: articles authored by individuals with surnames appearing earlier in the alphabet were more likely to be cited. This effect was especially pronounced in the context of alphabetical citation systems, compared with numerical citation systems. The current research provides a testable, reliable explanation for the surname order bias and establishes a causal link between surname alphabetical order and citation frequency. Implications for theory and academic practice are discussed.

**Supplementary information:**

The online version contains supplementary material available at 10.3758/s13423-025-02727-0.

How is a research article cited across academic contexts? This question holds significant interest and importance for cognitive, psychological, sociological, and information scientists (Nicolaisen, 2007). As a critical tool for scientific communication, citation also highlights the significance of publication as an institutionalized system that not only generates knowledge but also allocates rewards, thereby emphasizing the importance of reputation to the academic endeavor (Hyland, 2003). Indeed, citation indicators are largely regarded, though problematic, as one of the major measures representing the impact of research publications (Garfield, 1955), the cumulative impact of a scholar’s research output (Hirsch, 2005), the productivity and impact of a scholar (Schreiber, 2008), and the prestige of a journal (Van Der Pol et al., 2015).

Whether citation frequency is an “objective” measure remains an open question. Such a discussion began in 1980s (Cronin, 1984) and continues to this day (Dougherty et al., 2024). As Aksnes et al. (2019) suggested, research quality is a multidimensional concept, highly cited papers do not necessarily reflect that this research is innovatively groundbreaking and of high societal values and relevance. Rather, plenty of factors can potentially contaminate the objectivity and credibility of citations. For example, rhetorical citations (i.e., self-citation, journal self-citation, and citations of recently published studies; Hsiao & Chen, 2018; Safer & Tang, 2009), papers with shorter titles (Letchford et al., 2015), and the use of fewer mathematical equations (Fawcett & Higginson, 2012) comparatively but unreasonably receive more citations from peers.

Recent research also discovered a new bias on citation and reference that is due to surname order—namely, *surname order bias.* By using archival data such as “extracting the number of times that each of the articles was cited in Web of Science and calculated the mean number of citations for each letter” (Stevens & Duque, 2019, p. 1022), past studies offered statistical evidence to demonstrate the existence of surname order bias. They found that in journals with alphabetical citations (explained soon), the citation frequency moderately decreased across letters (*τ* = −0.34 [−0.56, −0.06], *BF* = 4.4). Their findings suggested that the number of citations was plausibly influenced by the alphabetical order of the first author’s surname, such that authors with surnames starting with an initial earlier in the alphabetical order (e.g., A, B, C) are cited disproportionately more than authors with surnames later in order (e.g., X, Y, Z).

However, the explanations provided by past studies for these results are “descriptive” instead of “mechanistic.” Following theories from cognitive psychology pertaining to human attention and memory, we propose two hypotheses to explain how citation and referencing behavior is biased by *surname alphabetical order*. Notably, the present research assumes that people’s automatic, reflexive responses—such as the widespread surname-order bias—override their reflective deliberations. So even scholars who recognize this bias will often continue to cite and reference under its influence. Additionally, Huang (2015) found that surname order bias primarily manifests when individuals cite others’ works, but not when citing their own. Therefore, we emphasize that the context explored in this research concerning surname order bias primarily pertains to external citations rather than internal citations.

## The selective exposure mechanism

With the limit of human attention, people cannot be fully exposed to all information in the environment and attend to all available information to the same degree. This notion stems from the fact that people have limited capacity for attention to information processing (Baddeley, 1992, 2012; Simon, 1991). Information that is exposed earlier (vs. later) is more likely to be available in people’s awareness. When it comes to citing and referencing behaviors, scholars may scan through the reference list and select the work coming earlier in the list due to limited time and attention (Stevens & Duque, 2019).

Generally, scholars’ and researchers’ citing/referencing behaviors are instructed by two commonly seen citation systems: alphabetical versus numerical. Specifically, the *alphabetical citation system* lists in-text citations and references according to the alphabetical order of the first author’s surname (e.g., APA style; see https://apastyle.apa.org/style-grammar-guidelines/references/examples/journal-article-references); whereas the *numerical citation system* organizes in-text citations and references based on the order of occurrence, utilizing numbers in the text to represent the order in the reference list (e.g., [1, 2, 3] in ACS style; see https://library.viu.ca/citing/vancouver).

Researchers usually check in-text citations following the order of the citation occurrence. Under the alphabetical citation system, the papers earlier in the order are more likely to be scanned earlier as well. Under the numerical citation system, however, the in-text citation order is not determined by the surname order. Moreover, when checking the reference list, researchers usually start from the earlier entry, which is also the earlier surname order under the alphabetical (but not the numerical) citation system. Thus, the order of information exposure and the surname order covary in the alphabetical citation system. People will be more likely to be attentive to papers associated with earlier surname orders. Ideally, this kind of differential attention will not happen in the numerical citation system. Yet we often notice some cases where papers are coincidentally placed alphabetically in the numerical citation system due to personal habit. Based on the reasonings above, the citation system will be a significant boundary factor. Accordingly, we hypothesize the following:

- **Hypothesis 1:** There will be a significant negative effect of surname alphabetical order on the citation frequency (preregistered).
- **Hypothesis 2:** There will be a significant interaction effect between the citation system (i.e., alphabetical vs. numerical) and surname alphabetical order on citation frequency, such that the negative effect of surname alphabetical order on citation frequency will be reduced or even eliminated under the numerical citation system (preregistered).

## The selective retrieval mechanism

The selective retrieval notion suggests that some information is more likely to be retrieved from our mental database than other information. Memory research indicates that the ease of memory retrieval and the mental accessibility of a piece of information depends on the familiarity, as indicated by its occurrence frequency, of that information (Gorman, 1961; Schulman, 1967). That is, the more familiar the information is, which happens when the information occurs more frequently, the more likely people are to retrieve it from memory.

Although there are 26 alphabet letters in English, their alphabetical order does not appear equal amounts of time in an English text (Yadav et al., 2010). Rather, letter frequency decreases along with the increment of alphabetical order (Lewand, 2000). Similarly, Norvig (2013) found that the letter frequency and the probability of being the first letter of words are negatively related, *r*(24) = −.22. According to the Decennial Census survey from the United States Census Bureau (https://www.census.gov/data.html), the population frequency of surnames starting with initials “A,” “B,” and “C” occupies about 22%, far more than that of surnames starting with initials “X,” “Y,” and “Z” (occupies only 1.2%).

Therefore, the letter frequency is expected to be negatively related to familiarity. If more frequently encountered names are more accessible and hence readily accessible in memory retrieval, we expect the following observations. First, the initial frequency should be positively related to the citation. That is, the more frequent the surname initial is (i.e., higher initial frequency), the more familiar it is to the people, and so the more likely it is to be cited. Second, surname alphabetical order should negatively influence citation frequency because the higher the surname order, the less frequent and less familiar, and eventually less likely to be cited. Third, if familiarity is the mechanism responsible for the surname order bias, the relationship between surname alphabetical order and citation frequency is expected to be mediated by initial frequency.

- **Hypothesis 3:** There will be a positive effect of initial frequency on citation frequency (preregistered).
- **Hypothesis 4:** There will be negative mediating effect of initial frequency on the relationship between surname alphabetical order and citation frequency (preregistered).

In short, people are expected to be more likely to cite a paper that they are attentive to (as a matter of attention), that they remember, and that is easily retrievable from memory (as a matter of memory).

## Overview

This research aims to investigate the influence of surname order bias on citation frequency and to determine whether this bias is specific to the citation system within various contexts. If the above is established, this study will also introduce a testable, reliable, and consistent explanation(s) for the observed surname order bias. Moreover, this research is of research and practical importance as it addresses the urgent call in the literature for a comprehensive understanding of the meaning and implications of citation practices, and how citation systems can be adjusted to provide a more accurate indication and smoother dissemination of research impact (Redner, 2005; Van Der Pol et al., 2015).

### Study 1

#### Method

##### Setting

In Study 1, we replicate and extend Stevens and Duque’s (2019) study by utilizing a larger sample size and controlling for additional factors, subsuming surname initial frequency and publication year. It is important and necessary to control for surname initial frequency because of the variance in surname initials distribution across ethnic groups. For instance, surnames beginning with “A” are more prevalent among Arab names (e.g., Abadi, Ali, Ahmad), while Chinese names have a higher proportion of surnames starting with “X,” “Y,” and “Z” (e.g., Xu, Yang, Zhang). Additionally, considering the number of nonnative English scholars (e.g., Chinese) seems to have exponentially increased in English-dominated academia in recent decades (Stockemer & Wigginton, 2019), controlling for the publication year is similarly vital and justified.

#### Procedure

We arbitrarily chose two disciplines that primarily use alphabetical citation systems (psychology and management) and two disciplines that primarily use numerical citation systems (biology and chemistry). We selected nine top journals from each of these four disciplines and compiled the total number of citations for each paper published in these 36 journals available in the Web of Science database from 1975 to 2017. We chose only top journals to ensure that the papers therein had a sufficiently large number of citations to enable us to test our hypotheses. Within each discipline, we arbitrarily chose three journals in three sub-categories. We are familiar with journals in psychology and management. The journals in these disciplines were chosen because we were aware that they represented the most important journals in their respective subcategories. We are less familiar with biology and chemistry journals. We chose the journals in these two disciplines through subjective screening, based on three criteria: (a) total number of citations (i.e., each journal was required to have a sufficiently large number of citations), (b) impact factor, and (c) normalized Eigenfactor score. The selected journals, along with their 2-year impact factors, normalized Eigenfactor scores, and number of articles included in the data analysis, are listed in Table 1.^1^

**Table 1:** Journals included in Study 1

Discipline	Journal Name	Impact Factor 2016	Normalized Eigen-factor Score	Number of Articles
Psychology: General	*Psychological Bulletin*	16.793	3.1443	2,632
	*Psychological Review*	7.638	1.2838	1,639
	*Psychological Science*	5.667	6.568	4,342
Psychology: Experimental	*Journal of Experimental Psychology:*			
	*General*	4.42	2.3752	1,898
	*Human Perception and Performance*	2.287	1.4758	4,137
	*Learning, Memory, and Cognition*	2.667	1.4768	4,169
	*Animal Learning and Cognition*	2.132	0.2151	1,549
	*Cognition*	3.414	2.8157	3,514
	*Journal of Memory and Language*	3.065	1.0575	2,406
Psychology: Social	*Journal of Personality and Social Psychology*	5.017	4.0639	7,741
	*Journal of Experimental Social Psychology*	2.159	2.112	3,029
	*Personality and Social Psychology Bulletin*	2.504	1.9518	4,796
Management: General	*Academy of Management Journal*	5.906	3.166	2,807
	*Academy of Management Review*	9.408	1.4682	2,287
	*Journal of Management*	7.733	2.5955	1,767
Management: Micro	*Journal of Applied Psychology*	4.13	2.2849	4,278
	*Organizational Behavior and Human Decision Processes*	2.454	1.0842	2,413
	*Personnel Psychology*	4.362	0.7133	4,559
Management: Macro	*Administrative Science Quarterly*	4.929	0.7868	2,511
	*Organization Science*	2.691	2.7562	1,630
	*Strategic Management Journal*	4.461	2.4329	2,630
Biology: Biochemistry	*Nucleic Acids Research*	10.162	42.9928	42,699
	*Nature Medicine*	29.886	20.4725	9,999
	*Genome Research*	11.922	13.0892	4,459
Biology: Cell	*Cell*	31.957	68.1441	19,616
	*Cancer Cell*	27.407	11.7944	2,523
	*Molecular Cell*	14.714	21.1223	6,841
Biology: Genetics/Heredity	*Nature Reviews Genetics*	40.282	11.7499	3,076
	*Nature Genetics*	27.959	27.7047	7,717
	*Trends in Ecology and Evolution*	15.268	4.5021	4,649
Chemistry: General	*Chemical Reviews*	47.928	28.2577	5,112
	*Chemical Society Reviews*	38.618	32.5744	4,583
	*Journal of the American Chemical Society*	13.858	88.454	126,406
Chemistry: Analytical	*Analytical Chemistry*	6.32	18.0166	43,224
	*TrAC Trends in Analytical Chemistry*	8.442	2.186	4,531
	*Sensors and Actuators B: Chemical*	5.401	8.1263	20,156
Chemistry: Inorganic/Nuclear	*Inorganic Chemistry*	4.857	12.4758	49,569
	*Dalton Transactions*	4.029	11.7473	20,170
	*Coordination Chemistry Reviews*	13.324	3.9383	4,691

As outlined in our pre-registration, we identified four disciplines, 36 journals, and a specific sample period prior to data collection. Notably, among these 36 journals, four overlap with the research conducted by Stevens and Duque: *Psychological Bulletin, Psychological Review, Psychological Science,* and *Journal of Experimental Psychology*. Consequently, this study does not represent a fully independent replication of Stevens and Duque’s work.

#### Sample and data collection

The data were collected between February 17, 2018, and February 20, 2018. We obtained all of the data from the Web of Science platform with a sample period from 1975 to 2017, using a Visual Basic program. We chose this period because the Web of Science platform at our institutes provided data from 1975 onward. We included all kinds of articles (e.g., reports, commentaries, editorials) in our analyses, ensuring that there were no missing articles. In total, we collected 446,755 articles, of which 66,734 were from the two disciplines with alphabetical systems and 380,021 were from the two disciplines using numerical systems.

#### Research design

We analyzed the data in two ways. First, the *surname initial* was used as the unit of analysis, giving a total of 26 data points. Second, we used the *research paper* as the unit of analysis, yielding 446,755 data points in total. Each of these two methods of analysis served as a robustness check for the other, although they had slightly different implications. Using the surname initial as the unit of analysis implied that the findings, if significant, would be generalizable across surnames. Using the research paper as the unit of analysis implied that the findings, if significant, would be generalizable across papers. We describe the two analyses separately below.

##### Surname initial as the unit of analysis

Taking the surname initial as the unit of analysis, we aggregate all articles by the initial of each surname, resulting in a total of 52 cases. Each citation system condition comprises 26 cases, with “A” representing 1 and “Z” representing 26. Notably, prefixes (e.g., al, de, van, von) are also considered as the first initial in our analysis. There were two independent variables: surname alphabetical order (1 to 26) and surname initial frequency (i.e., the frequency of each surname initial in our dataset). Surname initial frequency was included to control for the influence of the natural frequency of occurrence of letters, which has been characterized to be the significant confounding of surname alphabetical order (Jaekel, 1997; Shevlin & Davies, 1997).

For each data point, we obtained the average number of citations and the average number of citations after controlling for *publication year*. We constructed two dependent variables. First, considering the accumulative nature of citation frequency, we log-transformed (natural log) the number of citations of each article (log (*N* +1)) to reduce the problem of skewness. For each of the 26 data points for surname alphabetical order, we computed the average number of log citations. We called this dependent variable Mean Log Cite. For example, of the 446,755 articles published from 1975 to 2017, 16,709 were written by authors whose surnames started with the letter “A.” The sum of the log citations for these 16,709 articles was 55,601.61. Thus, the Mean Log Cite for “A” was 3.328 (i.e., 55,601.61/16709). With one Mean Log Cite value for each initial letter, there were 26 values of Mean Log Cite in total.

Second, we suspected that many of the authors with surnames beginning with “X,” “Y,” or “Z” (e.g., Xu, Xiao, Yu, Yao, Zhao, Zheng, and Zhu) were Chinese. The community of Chinese scholars has experienced relatively recent growth, implying that much of the research associated with surnames late in the alphabet was published only very recently, with relatively little time in which to accumulate citations. To control for this possible confounding factor, we controlled for the year of publication by standardizing the log cite to a *z*-score within each year (i.e., the mean number of citations for each year was 0 and its standard deviation was 1). After this standardization, the mean and standard deviation could not vary across publication years. This eliminated the influence of the year of publication. After the *z*-score transformation, we computed the mean of the *z*-scores of the log cite values, following the same method used to obtain the Mean Log Cite. We called this dependent variable Mean Z Log Cite (Table 2).

**Table 2:** The exact values of each data point in Figure 1 and Figure 2 in the main text in Study 1

Surname Initial	Initial Frequency	Overall Data			Alphabetical			Numerical	
		Mean Log Cite	Mean Z Log Cite	Initial Frequency	Mean Log Cite	Mean Z Log Cite	Initial Frequency	Mean Log Cite	Mean Z Log Cite
A	16,709	3.3276	1.2023	2,270	3.2868	1.1899	14,439	3.3341	1.2042
B	35,498	3.3123	1.1976	6,072	3.1278	1.1403	29,426	3.3504	1.2091
C	31,823	3.3132	1.1979	4,286	3.1170	1.1369	27,537	3.3437	1.2071
D	19,727	3.3043	1.1952	3,030	3.2640	1.1829	16,697	3.3116	1.1974
E	7,066	3.3906	1.2210	1,235	3.4545	1.2397	5,831	3.3770	1.2170
F	14,309	3.3067	1.1960	2,581	3.1784	1.1564	11,728	3.3349	1.2045
G	22,057	3.3266	1.2020	3,616	3.2745	1.1862	18,441	3.3369	1.2050
H	26,575	3.3427	1.2068	4,329	3.1445	1.1456	22,246	3.3812	1.2182
I	4,398	3.4285	1.2321	458	3.3717	1.2154	3,940	3.4351	1.2340
J	9,655	3.3020	1.1945	1,566	3.2319	1.1731	8,089	3.3155	1.1986
K	26,545	3.3086	1.1965	3,825	3.0736	1.1229	22,720	3.3481	1.2084
L	29,051	3.3029	1.1948	3,684	3.0965	1.1303	25,367	3.3329	1.2039
M	33,425	3.2657	1.1835	5,824	3.1172	1.1369	27,601	3.2970	1.1930
N	10,390	3.3424	1.2067	1,240	3.3645	1.2133	9,150	3.3394	1.2058
O	7,319	3.3715	1.2153	999	3.1925	1.1608	6,320	3.3997	1.2237
P	19,028	3.2924	1.1916	2,926	3.2098	1.1662	16,102	3.3074	1.1962
Q	1,452	3.2752	1.1864	97	3.0881	1.1276	1,355	3.2885	1.1904
R	17,815	3.3307	1.2032	3,305	3.1790	1.1566	14,510	3.3653	1.2135
S	43,603	3.2869	1.1899	7,103	3.1025	1.1322	36,500	3.3228	1.2008
T	16,088	3.4024	1.2245	2,191	3.2064	1.1652	13,897	3.4333	1.2335
U	1,704	3.3835	1.2189	242	2.9661	1.0872	1,462	3.4526	1.2391
V	7,808	3.2825	1.1886	1,362	3.1297	1.1409	6,446	3.3148	1.1984
W	21,919	3.3006	1.1941	3,149	3.1825	1.1577	18,770	3.3204	1.2001
X	2,749	3.2897	1.1908	60	2.7647	1.0169	2,689	3.3014	1.1944
Y	8,563	3.3660	1.2137	531	2.8861	1.0599	8,032	3.3978	1.2231
Z	11,479	3.2509	1.1789	753	3.1200	1.1378	10,726	3.2601	1.1817

**Fig. 1:**
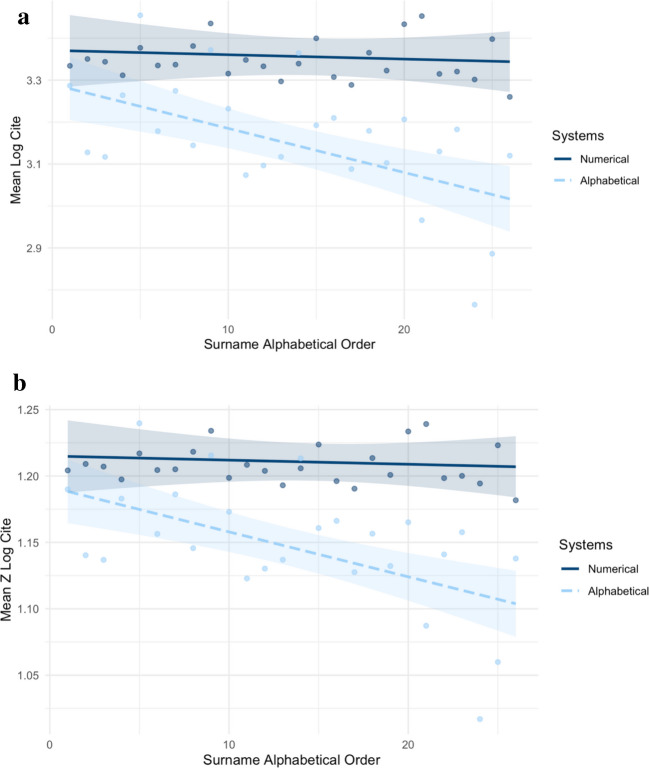
**a** The effects of surname alphabetical order on Mean Log Cites by citation systems, using surname as unit of analysis in Study 1. **b **The effects of surname alphabetical order on Mean Z Log Cites by citation systems, using surname as unit of analysis in Study 1

**Fig. 2:**
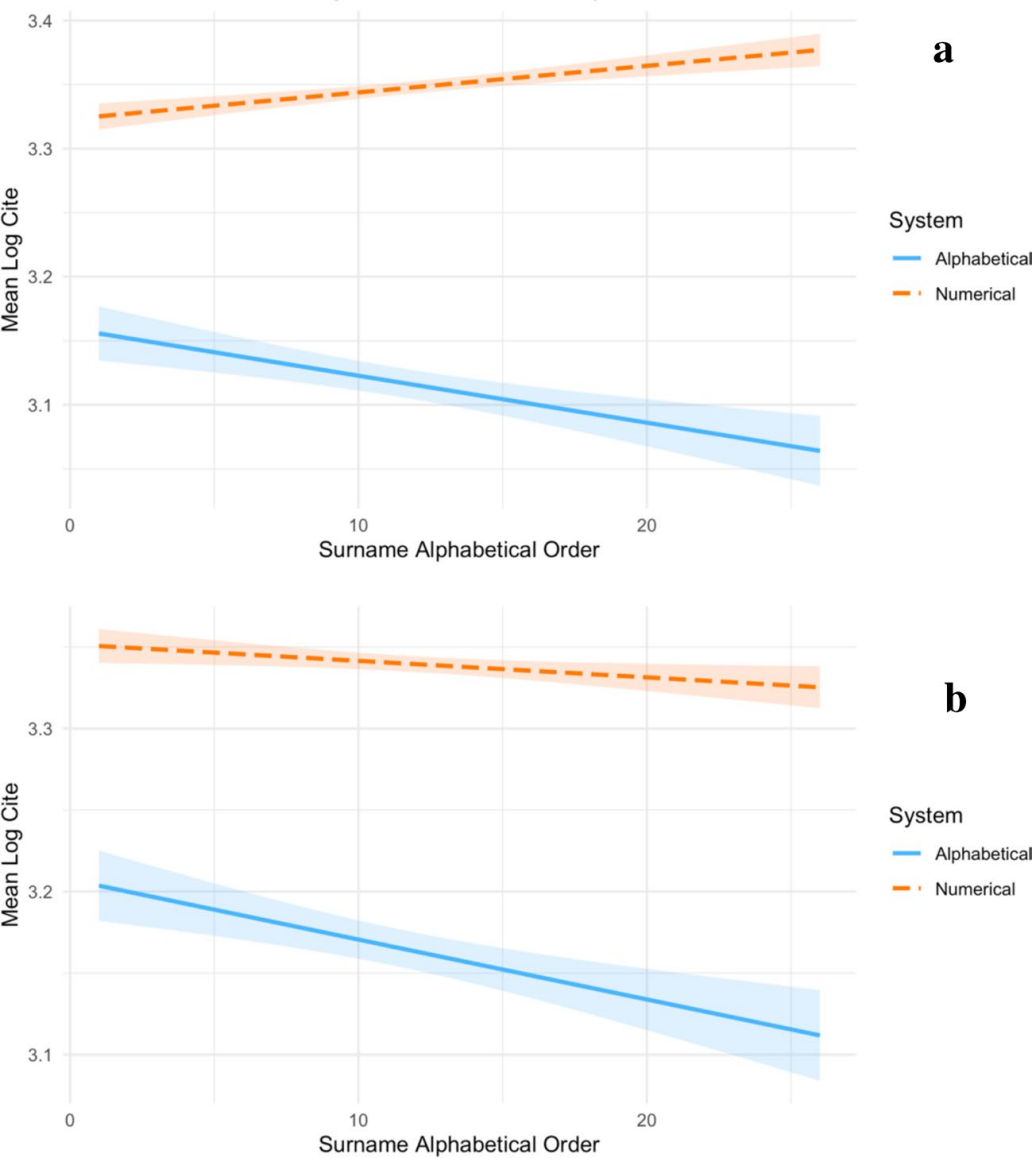
**a** The effects of surname alphabetical order on Mean Log Cites by citation systems controlling for publication years, using article as unit of analysis in Study 1. **b.** The effects of surname alphabetical order on Mean Log Cites by citation systems without controlling for publication years, using article as unit of analysis in Study 1

##### Research paper as the unit of analysis

Taking the research paper as the unit of analysis, the total number of data points was 446,755, corresponding to the total number of articles included in the analysis. Three independent variables were included: surname alphabetical order, surname initial frequency, and citation system. In addition, we included the publication year (from 1975 to 2017) as a control variable. Therefore, for each data point, we had one number representing surname alphabetical order (i.e., 1 for “A” and 26 for “Z”), one number representing surname initial frequency, one number representing citation system (0 = alphabetical, 1 = numerical), and one number representing Publication Year. The analysis involved only one dependent variable: the log-transformed number of citations (i.e., log (*N* +1)). We performed two sets of analyses: one with the control variable and one without it.

A sensitivity analysis conducted in G*Power software in Version 3.1 revealed that the final sample of 446,775 (α = .05, 99% power) for a test of five predictors can detect the minimum effect *f*^2^ = 5.98^e-05^.

#### Study 1: Results

Regression analyses with the surname initial as the unit of analysis (Table 3) revealed a negative effect of surname alphabetical order on both Mean Log Cite and Mean Z Log Cite (*B* values ≤ −.307, *p* values = .003), supporting our Hypothesis 1. This was a large effect (η_p_^2^ = .18), implying that approximately 18% of the variance in number of citations can be attributed to the surname alphabetical order, after controlling for system, initial frequency, and their interaction. Additionally, the results also manifested a significant interaction effect of citation system and surname alphabetical order on both Mean Log Cite and Mean Z Log Cite (*p* values ≤ .010, η_p_^2^ ≥ .13). When divided by citation systems, the results revealed that under the alphabetical citation system, surname alphabetical order negatively predicted Mean Log Cite and Mean Z Log Cite (*B* values ≤ −.603, *p* values ≤ .003). These results suggested a large effect size (η_p_^2^ = .32), indicating that under alphabetical citation system, approximately 32% of the variance in number of citations can be attributed to the surname alphabetical order after controlling for other factors. However, no significant effects were found under the numerical citation system (*p* values > .510, η_p_^2^ = .02). These results supported our Hypothesis 2. The moderating effects were plotted in Fig. 1. On the other hand, we found a negative and nonsignificant effect of initial frequency on both Mean Log Cite and Mean Z Log Cite (*B* values ≤ −.105, *p* values ≥ .390, η_p_^2^ *≤ .02*), not supporting our Hypothesis 3. According to Baron and Kenny’s (1986) three-step mediation framework, the mediating effect fails when the effect of the mediator on dependent variable is not present. Herewith, our Hypothesis 4 was not supported.

**Table 3:** Results of regression analyses using surname initial as unit of analysis in Study 1. *N* = 26 in all analyses.

	Mean Log Cite				Mean Z Log Cite			
	*B*	*SE*	*p*	*η*_*p*_^*2*^	*B*	*SE*	*p*	*η*_*p*_^*2*^
**Overall Data**								
Intercept	.000	.092	1.000	.00	.000	.092	1.000	.00
Surname Alphabetical Order (1 = “A” to 26 = “Z”)	-.307	.096	.003	.18	-.310	.097	.003	.18
System (0 = alphabetical, 1 = numerical)	.740	.127	.000	.42	.726	.128	.000	.41
Initial Frequency	-.114	.131	.390	.02	-.105	.132	.432	.01
Surname Alphabetical Order x System	.254	.095	.010	.13	.261	.096	.009	.45
**- Alphabetical System Data**								
(Intercept)	.0000	.168	1.000	.00	.000	.169	1.000	.00
Surname Alphabetical Order	-.610	.185	.003	.32	-.603	.185	.004	.32
Initial Frequency	-.167	.185	.375	.03	-.145	.185	.441	.03
**- Numerical System Data**								
(Intercept)	.000	.197	1.000	.00	.000	.197	1.000	.00
Surname Alphabetical Order	-.139	.213	.520	.02	-.142	.213	.510	.02
Initial Frequency	-.271	.213	.215	.07	-.269	.213	.219	.07

We also analyzed the same dataset with articles as the unit of analysis (i.e., 446,755 instead of 26 data points in total). The same pattern of interactions was found when we used articles as the unit of analysis (see Table 4). In this analysis, since our sample size is too large, using partial eta-squared may be meaningless (Fritz et al., 2012; Tomczak & Tomczak, 2014). Thus, we reported the 95% confidence intervals for the effect of each independent variable. Specifically, in overall data, the effect of surname alphabetical order on Mean Log Cite was significant irrespective of controlling Publication Year (*B* values = −.018, *p* values < .001), supporting Hypothesis 1. Next, the interaction of surname alphabetical order and citation system was highly significant (*p* values ≤ .003) regardless of controlling publication year or not. When divided by citation systems, with publication year as the control variable, we found that the influences of surname alphabetical order under the alphabetical system was negative (*B* = −.021, *p* < .001, 95% CI [−.031, −.011]), while the effect under the numerical system was positive (*B* = .012, *p* < .001, 95% CI [.008, .017]). These results were demonstrated in Fig. 2a. Without the Publication Year as the control variable, as demonstrated in Fig. 2b, we found that the negative influences of surname alphabetical order under the alphabetical citation system was stronger (*B* = −.021, *p* < .001, 95% CI [−.031, −.011]), compared to under the numerical citation system (*B* = −.004, *p* = .009, 95% CI [−.031, −.011]). These results supported our Hypothesis 2. In contrast, we found a negative effect of Initial Frequency on Mean Log Cite on both Models 1 and 2 (*B* values ≤ −.004, *p* values ≤ .068), not supporting our Hypotheses 3 and 4. All together, these results provided more robustness to support the selective exposure mechanism.

**Table 4:** Summary of regression analyses using article as unit of analysis in Study 1

	Model 1					Model 2				
	*B*	*SE*	*p*	*η*_*p*_^*2*^	95% CI	*B*	*SE*	*p*	*η*_*p*_^*2*^	95% CI
**Overall Data (*N* = 446,755)**										
Intercept	-.131	.004	.000	.00	[-.138, -.123]	-.099	.004	.000	.00	[-.107, -.092]
Surname Alphabetical Order (1 = “A” to 26 = “Z”)	-.018	.004	.000	.00	[-.026, -.009]	-.018	.004	.000	.00	[-.026, -.009]
System (0 = alphabetical, 1 = numerical)	.154	.004	.000	.00	[.146, .162]	.117	.004	.000	.00	[.108, .125]
Initial Frequency	-.005	.002	.014	.00	[-.009, -.001]	-.004	.002	.068	.00	[-.007, .000]
Publication Year	-.193	.002	.000	.04	[-.196, -.190]					
Surname Alphabetical Order x System	.027	.004	.000	.00	[.019, .036]	.013	.004	.003	.00	[.004, .021]
**- Alphabetical System Data (*N* = 66,734)**										
Intercept	.000	.004	1.000	.00	[-.008, .008]	.000	.004	1.000	.00	[-.008, .008]
Surname Alphabetical Order	-.021	.005	.000	.00	[-.031, -.011]	-.021	.005	.000	.00	[-.031, -.011]
Initial Frequency	-.014	.005	.007	.00	[-.023, -.004]	-.013	.002	.010	.00	[-.023, -.003]
Publication Year	-.135	.004	.000	.02	[-.143, -.128]					
**- Numerical System Data (*N* = 380,021)**										
Intercept	.000	.002	1.000	.00	[-.003, .003]	.000	.002	1.000	.00	[-.003, .003]
Surname Alphabetical Order	.012	.002	.000	.00	[.008, .017]	-.004	.002	.009	.00	[-.008, .001]
Initial Frequency	-.003	.002	.157	.00	[-.007, .001]	-.002	.002	.045	.00	[-.006, .003]
Publication Year	-.206	.002	.000	.04	[-.209, -.203]					

Mean Log Cite was the dependent variable. Publication Year was included as a control variable in Model 1 but not in Model 2. The most important finding was the significant interaction between Surname Order and Citation System, suggesting that the negative relationship between Surname Order and Mean Log Cite was stronger under the alphabetical system than under the numerical system

#### Robustness checks

Notably, the findings above were based on all publications from 36 journals. We did not consider the influence of publication types, such as reviews, comments, perspectives, and letters to the editors, as we believe that these considerations fall outside the scope of our research objectives. Conversely, introducing additional factors may introduce noise into this study. Nonetheless, for exploratory purposes, we filtered out non-research articles using keywords and utilized this subset of data (*N* = 432,891) to replicate the aforementioned regression analyses. The patterns of results remained consistent, with only minor differences in the coefficients. Interested readers can refer to our supplementary materials Table S1a and S1b.

Additionally, some readers may be skeptical about the current operationalizations of citation frequency and publication years. For instance, using a log-transformation method necessitates transformation and does not maintain the original scale of the citation frequency; publication years may be better modeled as a random effect rather than as a linear fixed effect; controlling for authors’ nationality would better mitigate potential confounders. Herewith, we employed the Poisson and negative Binomial regression models to replicate the analyses above. Interested readers can refer to our supplementary materials Tables S2–S7. All the results showed a same pattern of findings with those obtained described in the main context, affirming that our main conclusions remain valid.

#### Study 1: Discussion

We found that surname alphabetical order negatively influenced citation frequency only under disciplines using the alphabetical citation system but not disciplines using the numerical citation system. Unexpectedly, information familiarity as indicated by initial frequency tended to be negatively related to citation frequency. The results of Study 1 supported the selective exposure hypotheses but not supported the selective retrieval hypothesis. Our findings are consistent with previous studies (e.g., Stevens & Duque, 2019), but we provided stronger and more robust evidence of surname citation bias because we analyzed a larger sample size and controlled for surname initial frequency and publication year.

### Study 2

#### Method

##### Settings

Notwithstanding that Study 1 validated the selective exposure mechanism, it still leaves questions about causality unanswered. While a reversed causality of our hypotheses is not logically possible because the surname of a scholar happened a long time before the citation. Nonetheless, we cannot confirm the causality of the relationship, which can only be obtained from studies that manipulated surname order. Therefore, in Study 2, we aimed to test the causal effect of surname order bias on citation by conducting a well-controlled experiment with manipulations of surname alphabetical order and citation systems.

Furthermore, the issues of surname frequency and the mental distinctive effect of surname are noticeable from study 1, and a well-manipulated experiment can help resolve them. We believed that Study 2 could help reconcile the mixed findings about the effect of surname order bias through a well-manipulated experiment (e.g., Abramo & D’Angelo, 2017; Huang, 2015; Shevlin & Davies, 1997; Stevens & Duque, 2019; Tregenza, 1997; Weber, 2018), further facilitating the current literature to establish a consensus about the effect of surname alphabetical order.

Lastly, the investigation into whether surname order bias influences the selection of in-text citations from multiple references in parenthesis remains underexplored. This question is both significant and intriguing, particularly in light of some journals’ restrictions on the number of allowable citations. Prominent scientific journals such as *Science* and *Nature* impose a cap, typically no more than 50 main-text references. Given these constraints, scholars are often compelled to make selective decisions about which citations to include when multiple works are comparably relevant. This decision-making process might systematically favor certain works based on factors such as the alphabetical order of authors’ surnames, potentially leading to a surname order bias. The limited number of citations allowed by some journals exacerbates this issue, as it forces researchers to be even more selective, thereby possibly magnifying the effects of any existing biases in citation practices.

#### Participants and procedure

We recruited 318 participants with PhD training from Prolific, of whom 155 were men. We only recruited participants with graduate above or equivalent educational degrees in order to ensure that our participants could comprehend an academic article and would be familiar enough with citation and referencing procedures. Supported by a priori power analysis, a sample size of *N* = 270 could yield 99% power to detect a small to medium effect size (*f* = 0.20) in a repeated-measure within and between interaction analysis of variance (ANOVA). We targeted a larger sample size of 318 fitting our prescreen standards to account for an expected 15% attrition rate.

The participants were asked to read a hypothetical article on teamwork skills development and then summarize its content in at least 200 words. This article was based on two published papers (Donia et al., 2018; Vogler et al., 2018), with some modifications to ensure that the respondents were not confused by complex theories and concepts. For instance, we rephrased some complicated sentences with simpler sentences and gave explanations for some uncommon terminologies. To understand the implications of the surname order bias in regard to its influences on the visibility and perceived credibility of scholarly work in in-text citation selection practice, we created 28 artificial references in the hypothetical article. Among those artificial references, there was one single citation and nine sets of multiple citations, and all the multiple citations were a trio.^2^ We asked our participants to include five to ten references in their summary, although we did not put an upper limit on the number of references that they could use. To rule out the surname frequency issues and mental distinctive effect on surnames of artificial references (i.e., people may be attentive and memorize distinctive surnames), we only considered commonly seen surnames documented in the Decennial Census survey from the USA Census Bureau. Thus, all hypothetical surnames are six characters in length and with two vowels (Table 5).

**Table 5:** Materials used in the Study 2

Order of in-text Citation	Version 1				Version 2				Version 3			
	Surnames	Alphabetical Order	Citation Frequency (Alphabetical)	Citation Frequency (Numerical)	Surnames	Alphabetical Order	Citation Frequency (Alphabetical)	Citation Frequency (Numerical)	Surnames	Alphabetical Order	Citation Frequency (Alphabetical)	Citation Frequency (Numerical)
1	ARCHER	2	.4909	.2766	HOWELL	11	.1698	.2745	PAYTON	19	.1538	.2449
2	JENSEN	13	.2545	.3191	RHOADS	22	.1509	.2941	BOWERS	2	.3077	.4286
3	SOMERS	24	.1636	.2128	CORTES	4	.3396	.3725	KIMMEL	14	.2692	.4694
4	DUGGAN	6	.4364	.2979	LINDER	16	.0943	.2745	TALLEY	24	.1923	.1837
5	MONTES	17	.1273	.3404	VERNON	26	.1887	.3137	ECHOLS	6	.3654	.3469
6	WARNER	27	.1091	.2553	FLOREZ	8	.3774	.4118	NEWSOM	18	.3462	.3265
7	GAYTAN	9	.4000	.2979	OLDHAM	19	.2075	.2549	YANCEY	27	.2885	.1837
8	HOLMAN	11	.2909	.3404	PULLEN	20	.1887	.3529	AMBRIZ	1	.3269	.4286
9	ROBINS	22	.3818	.1915	BROOKS	2	.5283	.4118	JEWELL	12	.2692	.2449
10	COLVIN	4	.4909	.3191	KELLER	14	.2075	.2353	SEXTON	22	.2500	.1429
11	LOWERY	15	.1091	.1915	TURNER	24	.0755	.1569	DUPONT	5	.2115	.1633
12	VALDES	26	.1091	.2553	ELKINS	6	.2642	.3725	MILLAN	16	.2115	.2449
13	FRASER	8	.5273	.4043	NUGENT	18	.2075	.1961	WHALEN	26	.1923	.3061
14	OSBORN	19	.0909	.1064	ZELLER	28	.1132	.1373	GRABER	9	.2885	.3265
15	POTTER	20	.0727	.1277	AHRENS	1	.4340	.1765	HOLTON	10	.2308	.1837
16	BENNER	3	.3091	.0638	JANSEN	13	.0943	.1373	ROWLEY	21	.1730	.1224
17	KELLEY	14	.1091	.1064	STREET	23	.0943	.1765	CLARKE	4	.2308	.3469
18	THOMAS Jr.	25	.0545	.0426	DURHAM Jr.	5	.1698	.0784	LACKEY Jr.	15	.0769	.1429
19	EMMONS	7	.3091	.2766	MOSLEY	17	.2075	.2745	VICTOR	25	.2115	.2041
20	NGUYEN	18	.2545	.2340	WATSON	27	.1887	.3333	FARRAR	8	.4038	.2653
21	QUALLS	21	.2364	.2340	GIBSON	9	.3208	.2353	INGRAM	11	.1731	.2449
22	ANDERS	1	.4182	.3830	HASSAN	10	.3774	.4118	PORRAS	20	.3654	.2041
23	JACOBS	12	.2182	.2340	RANSOM	21	.3208	.1765	BREWER	3	.4615	.4286
24	SAWYER	23	.3273	.3404	COBURN	3	.6038	.3333	KENYON	13	.4808	.4694
25	DOWELL	5	.5091	.2340	LARSEN	15	.2830	.2549	TALBOT	23	.3654	.4082
26	MONSON	16	.4364	.4043	VALDEZ	25	.4340	.5294	ECKERT	7	.5577	.4286
27	WILLEY	28	.2364	.2340	FISHER	7	.5660	.3725	NEGRON	17	.3654	.2653
28	GRIMES	10	.4909	.5106	IRVLNG	12	.3585	.3725	YBARRA	28	.5192	.3878

The participants completed the task at times and places convenient to them. We did not set a time limit. On average, they took 60.70 minutes to complete the task. The article was in PDF format with a bookmark function. As in conventional online articles, the bookmark function directed the participants to the reference list at the end of the article when they clicked on the in-text citations. An average of 2.8 days after completing the task, the participants received a debriefing message explaining to them that the citations and references in the article had been artificially created for research purposes. Data from 11 participants were excluded because they failed to meet the study requirements, such as by providing fewer than five citations and/or a summary with fewer than 200 words. The analyses described below were conducted with the data from the remaining 307 participants.

#### Experimental design and materials

This experiment included a two-level between-participants factor (i.e., Citation System: alphabetical vs. numerical) and a three-level within-participants factor (i.e., Reference Alphabetical Category: early, middle, or late).

The Citation System condition was manipulated by randomly assigning participants to either the alphabetical citation system or the numerical citation system. This manipulation involved presenting the same article with citations formatted according to two different styles: APA (alphabetical) and Vancouver (numerical). It is important to note that in our material design, the display orders of the in-text citations were identical but only the in-text citation formats were dissimilar for both articles of alphabetical and numerical system conditions. For instance, assuming that the first set of multiple citations is from “Archer’s 2013 work, Jensen’s 2008 work, and Somers & Jones’s 2011 work”; in alphabetical system condition, the in-text citations were presented as “(e.g., Archer, 2013; Jensen, 2008; Somers & Jones, 2011)” whereas, in numerical system condition, it is “[1, 2, 3].”

Such a deliberative design (i.e., identical display order but dissimilar format) was chosen for two reasons. First, the manipulation of the first authors’ surnames in a hypothetical article of numerical system condition renders it almost impossible to establish a convincing and appropriate basis for ordering these surnames in a naturalistic manner. Even employing a random sorting method for the surnames could complicate the counterbalancing process within the experimental design and introduce unexplained variables. Second, in practical circumstances, we can often observe the cues of alphabetical order presented within in-text citations under numerical system’s style requirements. This occurrence can be attributed to 1) personal habits, and 2) the functionality of the citation machine. Scholars accustomed to a particular citation style may inadvertently maintain elements of that style out of habit, even when guidelines require a different format. Likewise, the processing algorithms of citation management tools can play a significant role. For instance, when a scholar initially uses APA format and later needs to switch to the Vancouver style for a manuscript resubmission, the citation software might simply adjust the citation format while retaining the alphabetical order of the authors. This is because many citation tools are programmed to prioritize ease of use and continuity in document preparation, which can lead to the retention of alphabetical ordering irrespective of the citation style specified. Thus, the blend of personal citation habits and the operational characteristics of citation software contributes to the persistence of alphabetical cues in numerical citation systems. In sum, we believe that building the alphabetical surname order bias in both citation systems in our experiment can offer a stronger test of the citation format.

Building on these, we believe that maintaining a consistent ordering as used in the articles of alphabetical system condition can help us better isolate and quantify the impact of surname order bias. Accordingly, we expect that the results from both citation system conditions should be comparable; however, the surname order bias is anticipated to be more pronounced in the alphabetical system condition than in the numerical system condition.

Furthermore, regarding the within-subject factor, the Reference Alphabetical Category was manipulated by classifying the 28 references in the article into three categories (early, middle, or late orders). Specifically, references were classified as follows: surnames starting with the letters *A* to *G* were allocated to the early order category, surnames beginning with *H* to *O* were designated as the middle order category, and those starting with *P* to *Z* were assigned to the late order category. This categorization approach aimed to ensure a balanced distribution across the categories, aligning with the distribution of common 5,000 surnames as reported in the Decennial Census survey by the USA Census Bureau, where the surnames were divided into 1,878 (37.5%) in the early order, 1,562 (31.3%) in the middle order, and 1,559 (31.2%) in the late order.

To counterbalance the positions at which the references appeared in the articles, we created three versions (Versions I to III) of each article by rotating the three categories, resulting in six articles (Versions I to III under the alphabetical system and Versions I to III under the numerical system). Specifically, 10 references belonging to the early order category in Version I were allocated to the middle order category in Version II and the late order category in Version III. Nine references belonging to the middle order category in Version I were reassigned to the late order category in Version II and to the early order category in Version III. Finally, nine references belonging to the late order category in Version I were reassigned to the early order category in Version II and to the middle order category in Version III. For example, a single in-text citation was “Grimes et al. (2005)” in Version A, “Irving et al. (2005)” in Version B, and “Ybarra et al. (2005)” in Version C. The participants were randomly assigned to read one of the three versions of each article. The materials used are summarized in Table 5. Interested readers can refer to Fig. S1 in the supplementary materials for the visualization of our experiment design.

##### Dependent variables

The dependent variables were the citation frequency for the three reference alphabetical categories. We classified all of the references for each participant into one of the three categories of alphabetical order. Accordingly, we obtained three citation frequencies for each participant, corresponding to the three Reference Alphabetical Category conditions. For example, one participant, who read Version I articles, cited three of the 10 references from the early order category, zero of the nine references in the middle order category, and two of the nine references in the late order category. The citation frequencies for this participant in the three order category conditions were .3, 0, and .222, respectively.

Table 6 shows the means and standard deviations for the citation frequency in all conditions. A 3 (Reference Alphabetical Category: early, middle, or late) × 2 (Citation System: alphabetical or numerical) × 3 (Counterbalance: Version I, Version II, or Version III) repeated-measure ANOVA was conducted to examine the predicted interaction between reference alphabetical category and citation system. A sensitivity analysis in G*Power revealed that the final sample of 318 (α = .01, 99% power) for a test with 18 groups and three measurements can detect the smallest effect *f* = .17.

**Table 6:** Mean and standard deviation in Study 2

Reference Alphabetical	Overall			Alphabetical System			Numerical System		
Category	Mean	SD	*n*	Mean	SD	*n*	Mean	SD	*n*
Early	3.38	1.68	307	3.73	1.78	160	3.01	1.48	147
Middle	2.35	1.45		2.17	1.50		2.54	1.38	
Late	2.15	1.53		2.04	1.61		2.27	1.43	

#### Study 2: Results

The ANOVA results are presented in Table 7. Specifically, in overall data, the main effect of Reference Alphabetical Category was significant (*F* = 64.68, *p* < .001, η_p_^2^ = .18) The follow-up pairwise comparison tests revealed that the mean citation frequency in the early order category condition was significantly higher than that in the middle category order condition (*MD* = 1.03, *F* = 71.11, *p* < .001, η_p_^2^ = .19), and that in the late order category condition (*MD* = 1.23, *F* = 92.22, *p* < .001, η_p_^2^ = .24). The mean difference between the middle order category condition and the late order category condition was significant (*MD* = .20, *F* = 5.14, *p* = .024, η_p_^2^ = .02). The main effect of the Citation System was not significant (*F* = .16, *p* = .691, η_p_^2^ < .01).

**Table 7:** ANOVA Results in Study 2

*DV = Citation Frequency*	*df*	*F*	*MS*	*p-value*	*η*_*p*_^*2*^
***3 x 2 x 3 ANOVA: Overall Omnibus Comparison***					
Reference Alphabetical Category	2, 602	64.68	127.84	< .001	.18
Citation System	1, 301	.16	.15	.691	< .01
Article Versions	2, 301	1.15	1.11	.319	.01
Citation System * Article Versions	2, 301	1.15	1.03	.345	.01
Reference Alphabetical Category * Article Versions	4, 602	6.77	13.38	<.001	.04
Reference Alphabetical Category * Citation System	2, 602	12.79	25.28	< .001	.04
Reference Alphabetical Category * Citation System * Article Versions	4, 602	4.17	8.23	.002	.03
***-- Follow-up Mean Comparison Tests Under Alphabetical Citation System***					
Article Versions	2, 157	.79	.76	.454	.01
Reference Alphabetical Category	2, 314	58.83	137.05	< .001	.27
Reference Alphabetical Category * Article Versions	4, 314	8.48	19.76	<.001	.10
***-- Follow-up Mean Comparison Tests Under Numerical Citation System***					
Article Versions	1, 144	1.36	1.32	.261	.02
Reference Alphabetical Category	2, 288	13.20	21.01	< .001	.08
Reference Alphabetical Category * Article Versions	4, 288	1.72	2.74	.145	.02
**Omnibus Comparison: Early vs. Middle**					
Reference Alphabetical Category	1, 301	71.11	164.70	<.001	.19
Citation System	1, 301	1.96	2.29	.162	.01
Article Versions	2, 301	.20	.23	.818	.00
Citation System * Article Versions	2, 301	1.69	1.97	.186	.01
Reference Alphabetical Category * Article Versions	2, 301	7.04	16.30	.001	.05
Reference Alphabetical Category * Citation System	1, 301	18.71	43.32	<.001	.06
Reference Alphabetical Category * Citation System * Article Versions	2, 301	3.53	8.17	.031	.02
***-- Under Alphabetical Citation System***					
Article Versions	2, 157	1.41	1.69	.247	.02
Reference Alphabetical Category	1, 157	70.04	193.75	<.001	.31
Reference Alphabetical Category * Article Versions	2, 157	7.67	21.21	<.001	.09
***-- Under Numerical Citation System***					
Article Versions	2, 144	.46	.52	.634	.01
Reference Alphabetical Category	1, 144	8.87	16.19	.003	.06
Reference Alphabetical Category * Article Versions	2, 144	1.79	3.26	.171	.02
**Omnibus Comparison: Early vs. Late**					
Reference Alphabetical Category	1, 301	92.22	231.48	<.001	.24
Citation System	1, 301	4.00	4.56	.046	.01
Article Versions	2, 301	.79	.90	.455	.01
Citation System * Article Versions	2, 301	1.74	1.98	.178	.01
Reference Alphabetical Category * Article Versions	2, 301	9.66	24.26	<.001	.06
Reference Alphabetical Category * Citation System	1, 301	12.95	32.50	<.001	.04
Reference Alphabetical Category * Citation System * Article Versions	2, 301	5.90	14.81	.003	.04
***-- Under Alphabetical Citation System***					
Article Versions	2, 157	1.61	1.81	.203	.02
Reference Alphabetical Category	1, 157	73.40	226.13	<.001	.32
Reference Alphabetical Category * Article Versions	2, 157	11.15	34.35	<.001	.12
***-- Under Numerical Citation System***					
Article Versions	2, 144	.93	1.07	.399	.01
Reference Alphabetical Category	1, 144	21.01	39.67	<.001	.13
Reference Alphabetical Category * Article Versions	2, 144	2.50	4.72	.085	.03
**Omnibus Comparison: Middle vs. Late**					
Reference Alphabetical Category	1, 301	5.14	5.67	.024	.02
Citation System	1, 301	4.00	6.35	.046	.01
Article Versions	2, 301	5.77	9.15	.003	.04
Citation System * Article Versions	2, 301	2.06	3.26	.130	.01
Reference Alphabetical Category * Article Versions	2, 301	2.03	2.24	.133	.01
Reference Alphabetical Category * Citation System	1, 301	.70	.78	.402	.00
Reference Alphabetical Category * Citation System * Article Versions	2, 301	1.56	1.72	.212	.01
***-- Under Alphabetical Citation System***					
Article Versions	2, 157	4.99	8.68	.008	.06
Reference Alphabetical Category	1, 157	1.10	1.25	.297	.01
Reference Alphabetical Category * Article Versions	2, 157	3.26	3.72	.041	.04
***-- Under Numerical Citation System***					
Article Versions	2, 144	2.63	3.73	.076	.04
Reference Alphabetical Category	1, 144	4.87	5.17	.029	.03
Reference Alphabetical Category * Article Versions	2, 144	.23	.24	.798	.00

Most importantly, the interaction between the Reference Alphabetical Category and Citation System was significant (*F* = 12.79, *p* < .001, η_p_^2^ = .04). When divided by systems, the effect of reference alphabetical category was significant under the alphabetical system (*F* = 58.83, *p* < .001, η_p_^2^ = .27). Specifically, the follow-up mean comparisons tests revealed that the mean citation frequency in the early order category condition was significantly higher than that in the middle order category condition (*MD* = 1.56, *F* = 70.04,* p* < .001, η_p_^2^ = .31), and that in the late order category condition (*MD* = 1.69, *F* = 73.40, *p* < .001, η_p_^2^* =* .32). The mean difference between the middle order category condition and the late order category condition was not significant (*F* = 1.10, *p* = .297, η_p_^2^ = .01).

The effect of alphabetical order was also significant under the numerical system (*F* = 13.20, *p* < .001, η_p_^2^ = .08). The follow-up means comparisons tests revealed that the mean citation frequency in the early order category condition was significantly higher than that in the middle order category condition (*MD* = .47, *F* = 8.87, *p* = .003, η_p_^2^ = .06), and that in the late order category condition (*MD* = .74, *F* = 21.03, *p* < .001, η_p_^2^ = .13). The mean citation frequency in the early order category condition was significantly higher than that in the late order category condition (*MD* = .27, *F* = 4.87, *p* = .029, η_p_^2^ = .03).

**Fig. 3:**
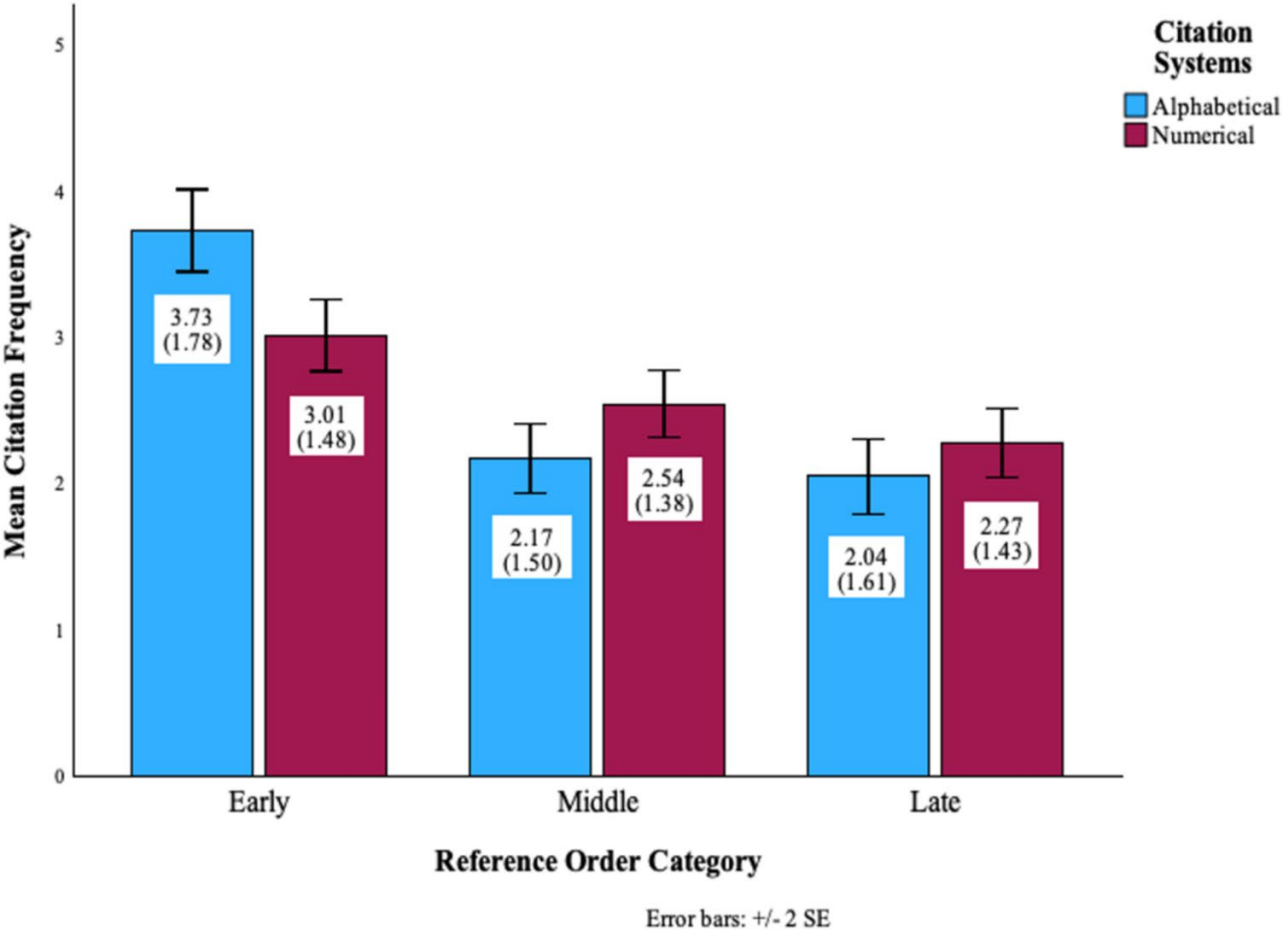
Mean Citation Frequency of Each Reference Order Category by Citation Systems in Study 2

The above results were visualized on Figure 3 and supplementary material Fig. S2. Although the simple effects of the reference alphabetical category were significant under both citation systems. The significant interaction between the reference alphabetical category and citation system meant that the effect of alphabetical order was greater under the alphabetical system, η_p_^2^ = .27 than under the numerical system, η_p_^2^ = .08.

#### Study 2: Discussion

The results of Study 2 were consistent with our expectation that the results from both citation system conditions were comparable, and the surname order bias should be more pronounced in the alphabetical system condition than in the numerical system condition. Accordingly, Study 2 provided support for the proposed causal links of the selective exposure mechanism. Our results revealed that (a) the selective exposure mechanism could be generalizable to the in-text citation decisions, and the surname alphabetical order was the cause of the observed citation bias, and (b) the surname order bias could be significantly reduced by replacing the alphabetical citation system with the numerical citation system.

## General discussion and conclusion

The present research contributes to the research evaluation literature by providing empirical support for the selective exposure mechanism, thereby addressing the theoretical ambiguity surrounding the impact of surname alphabetical order on citation frequency (Huang, 2015). Specifically, our findings demonstrated that individuals, regardless of early and contemporary scholars in disciplines utilizing alphabetical citation systems, tend to or are more habitually trained to pay closer attention to authors listed earlier in the reference list or in-text citation bracket when making decisions about citations.

Additionally, contrary to the possible assumption that citations are predominantly driven by familiarity with specific authors’ works, our data based on the initial frequency do not support this hypothesis. Since familiarity is a multifaceted construct and can be context dependent (Dougherty et al., 2024), our work focusing on the actual distribution of surname initials and their frequency provides an alternative lens in testing the selective retrieval mechanism on citation behaviors. These findings are important as they underscore the influence of cognitive biases in scholarly practices, where the position of an author’s name in the alphabet can inadvertently affect their visibility and citation frequency. Our research findings also challenge the conventional belief that citations are purely merit-based and highlights the need for awareness and possibly corrective measures in scholarly citation practices to ensure a fairer representation of contributions across the spectrum.

Furthermore, the current research significantly contributes to the research motivation literature by integrating an examination of specific academic characteristics and processes, such as citation systems and citation frequency, into the broader discourse on research performance determinants. Prior research has predominantly concentrated on the role of social-relational mechanisms, such as highlighting the impact of professional relationships and network centrality on early career development and performance in academia (Gersick et al., 2000; Hadani et al., 2012), the inbound mobility on research performance through a social learning and comparison mechanism (Slavova et al., 2016), and interdisciplinary collaboration to trade-off the publications and citations (Leahey et al., 2017).

However, past studies overlook the granular, yet crucial, aspects specific to the academic environment. That is, the mechanics of citation practices and their frequency. This oversight can potentially skew our understanding of research performance since citation metrics are a foundational element of academic recognition and influence. Therefore, this research responds to the calls for a more nuanced understanding of citation behaviors within academic contexts (Johns, 2006, 2017). By focusing on the intrinsic details of citation systems and their impact on research performance, this research not only broadens the existing theoretical framework provided by social-relational analyses but also enhances our understanding of the motivational factors that drive scholarly work. The inclusion of citation practices and their specific characteristics in academia offers a more comprehensive view of the factors that influence research performance, thereby enriching the theoretical landscape of research motivation literature.

We advise readers to interpret our findings with caution. Although the ethnicity of researchers and the ethnic connotations of their surnames fall outside the scope of this study, we recognize that these factors could potentially confound citation and referencing behaviors, thereby affecting our results. Accurately identifying the specific associations (e.g., stereotypes or biases) the researchers may hold regarding certain surnames during citation practices presents a significant challenge and is time-consuming. Accordingly, we encourage scholars interested in this area to consider the following directions for future research: (1) identify the implicit meanings associated with common surnames (e.g., Smith, Johnson for Europeans); (2) examine which meanings are relevant to citation behaviors; and (3) investigate whether these meanings can act as moderators in the relationship between surname alphabetical order and citation frequency.

Furthermore, we advise our readers to exercise caution when interpreting the selective retrieval mechanisms presented in the present research. Previous studies (e.g., Dougherty et al., 2024) indicates that the memory processes involved in citation decisions are complicated and nuanced. These processes are context-dependent and can evolve dynamically over time. Consequently, it is challenging to disentangle the effects of attention and familiarity in typical circumstances. While this research offers a framework for examining selective retrieval mechanisms based on initial frequency, we encourage future studies to explore how different priming and framing effects can be designed to elicit selective memory retrieval processes and examine how these processes develop over time.

To conclude, surname order bias is an important topic for future research since it can potentially disrupt communication, impede the dissemination of knowledge, and undermine the recognition of others academic endeavors. Our findings, along with Stevens and Duque, straightforwardly suggest that we should replace the alphabetical citation system with the numerical citation system. A deeper implication is to replace using citation as the proxy of the primary indicator research quality. We should consider the recommendations of Aksnes et al. (2019) to actively promote research quality as a multidimensional concept. Both journals and scholars must work to diminish the uncritical reliance on citations within the academic community. By doing so, citations will not be ascribed inappropriate or unreasonable meanings anymore, and it can also return to its intended purpose: acknowledging and giving credits to the contributions of prior research. By implementing the above suggested changes, we can collectively work towards a more balanced and substantiated scholarly communication environment, ultimately advancing the quality and equity of academic research.

## Supplementary information

Below is the link to the electronic supplementary material.

Supplementary file1 (DOCX 419 KB)

## Data Availability

The coding syntax is publicly available for review.

## Footnotes

1 Please note that the 36 journals of our research subjects all arrange the authorship order based on the actual contributions of the authors. Our samples do not include journals that determine authorships based on alphabetical order of the authors’ names (e.g., high-energy physics, computer science, mathematics). Excluding these journals should not bias our analysis and results for two main reasons. First, these fields still use alphabetical or numerical citation systems, with minor formatting differences, such as italics or bold for titles. In our analysis, citation frequency reflects individual articles, all authors gain visibility. It suggests our findings should not greatly differ in including or excluding the journals using different standards of authorship order. Rather, including those journals may probably enhance our results’ visibility, as readers should pay more attention to authors with earlier initials. Second, research in those fields areas often involves large collaborative projects, especially in high-energy physics, where dozens or even hundreds of scientists participate. The substantial volume of literature produced complicates the quantification and assessment of authors’ contributions due to the demands of rapid publication and large collaborations. Using surname’s alphabetical order as authorship order simplifies this process and reduces authorship disputes. Thus, we believe the primary audience for this paper may not be scholars from these fields.

2 For the detail of the materials please visit: https://osf.io/5wjsq/?view_only=f7e39da88800437b9faf8d6cd5e5e7fd

## Declarations

### Open practices statement

Following the American Psychological Association Journal Article Reporting Standards, all manipulations and measures and sample size determination were described in the following sections. All study materials, data, and analysis scripts were available on the Open Science Framework (OSF) page. Study 1 was preregistered on February 11, 2018 (https://osf.io/vwphk/?view_only=ba9eaa7903c449e2871a693a5efa3010). Study 2 was preregistered on January 15, 2023 (https://osf.io/5wjsq/?view_only=f7e39da88800437b9faf8d6cd5e5e7fd). In the preregistration process, we specified the hypotheses, sample size and composition, data collection process, data inclusion criteria, and plan for analysis. The data files and analysis syntax can also be accessed on OSF (for Study 1: https://osf.io/2thxs/files/osfstorage?view_only=bfb599cf09bd4c2fa673e12792ae82e9; for Study 2: https://osf.io/4r5av/files/osfstorage?view_only=3788e1e306884c84b0ab40c0adc3825a).
