# Supplementary material for: Does your surname undermine your research impact?
Source: Psychon Bull Rev. 2025 Aug 4;32(6):3116–33. doi: 10.3758/s13423-025-02727-0 (PMC12627127; doi:10.3758/s13423-025-02727-0)
Supplement: Supplementary file 1 — Supplementary file1 (DOCX 419 KB) [file 13423_2025_2727_MOESM1_ESM.docx]

**SUPPLEMENTARY ANALYSIS**

Robustness check for findings of study 1

***Excluding publications other than research articles.*** Notably, the findings reported in the main text were based on all publications from 36 journals. While these journals periodically publish other types of articles—such as comments, perspectives, and letters to the editor—the predominant publication type is research articles. We believe there is no theoretical need to exclude these alternative publication types, as they constitute a very small proportion of our sample. If there exists a relationship contradictory to our hypothesis in the other publication types, their impact on our research findings is expected to be minimal. Conversely, if there is a more pronounced surname alphabetical order effect on citation frequency within these alternative publication types, we would have even less justification for excluding them. Additionally, one more critical reason for not focusing solely on research articles by excluding other publications is that we do not have the time and labors to manually verify whether each paper is a research article, given our extensive sample size (over 400k). Therefore, we ultimately decided to include all published papers as part of our sample.

Additionally, to satisfy our research curiosity, we developed a program to help us automatically identify different publication types based on specific keywords in the titles. Specifically, we propose several common keywords for each type of publication.

- Review: "systematic review", "meta-analysis", "overview", "narrative review", "reviews"
- Comment: "comment", "response", "reply", "discussion", "rejoinder"
- Perspective: "perspective", "viewpoint", "opinion", "insight"
- Letter to the Editor: "letter", "correspondence", "editorial"

Through this method, we subsequently found 7,426 comments, 372 letters to the editor, 3,931 perspectives, and 2,135 reviews. The remaining 432,891 papers are research articles, and we then used this subset of data to replicate the analyses conducted in the main text.

As shown in Tables S1a, the interaction effect of citation system and surname alphabetical order on both Mean Log Cite and Mean Z Log Cite were significant (*ps* ≤ .016). When divided by citation systems, the results revealed that under the alphabetical citation system, surname alphabetical order was negatively predicted Mean Log Cite and Mean Z Log Cite (*Bs* ≤ -.567, *ps* ≤ .007). However, such an effect was not significant under the numerical citation system (*ps* ≥ .479). Similarly, as shown in Table S1b, the results using research articles as unit of analysis indicated that the interaction effect of surname alphabetical order and citation system was significant regardless of controlling publication year or not (*ps* ≤ .0495). When divided by citation systems, with publication year as the control variable, we found that the effect of surname order under the alphabetical system was negative (*B* = -.017, *p* = .001), while this effect under the numerical system was positive (*B* = .013, *p* < .001). Without the Publication Year as the control variable, we found that the negative influences of surname order under the alphabetical citation system was stronger (*B* = -.018, *p* = .001), compared to under the numerical citation system (*B* = -.004, *p* = .102).

In sum, these results showed a consistent pattern of findings, evidencing that our main conclusions still hold, and the publication types should not be a major concern in our research.

**Table S1a.** Summary of regression analyses using last name initial (N = 26) as the unit of analysis based on 432,891 research articles in Study 1.

|  | **Mean Log Cite** | | | |  | **Mean Z Log Cite** | | | |
| --- | --- | --- | --- | --- | --- | --- | --- | --- | --- |
|  | ***B*** | ***SE*** | ***p*** | ***η******_p_^2^*** |  | ***B*** | ***SE*** | ***p*** | ***η_p_^2^*** |
| **Overall data** |  |  |  |  |  |  |  |  |  |
| Intercept | .000 | .092 | 1.000 | .00 |  | .000 | .092 | 1.000 | .00 |
| Surname Alphabetical Order (A = 1 to Z = 26) | -.291 | .096 | .004 | .16 |  | -.295 | .097 | .004 | .17 |
| System (0 = Alphabetic, 1 = Numerical) | .739 | .128 | < .001 | .41 |  | .725 | .129 | < .001 | .40 |
| Initial Frequency | -.091 | .132 | .494 | .01 |  | -.083 | .133 | .536 | .01 |
| Surname Alphabetical Order x System | .238 | .095 | .016 | .12 |  | .245 | .096 | .014 | .12 |
|  |  |  |  |  |  |  |  |  |  |
| **Alphabetical System Data** |  |  |  |  |  |  |  |  |  |
| Intercept | .000 | .172 | 1.000 | .36 |  | .000 | .173 | 1.000 | .36 |
| Surname Alphabetical Order (A = 1 to Z = 26) | -.573 | .189 | .006 | -.18 |  | -.567 | .190 | .007 | -.17 |
| Initial Frequency | -.123 | .189 | .523 | .27 |  | -.102 | .190 | .596 | .29 |
|  |  |  |  |  |  |  |  |  |  |
| **Numerical System Data** |  |  |  |  |  |  |  |  |  |
| Intercept | .000 | .199 | 1.000 | .00 |  | .000 | .199 | 1.000 | .00 |
| Surname Alphabetical Order (A = 1 to Z = 26) | -.151 | .214 | .488 | .02 |  | -.154 | .214 | .479 | .02 |
| Initial Frequency | -.229 | .214 | .297 | .05 |  | -.226 | .214 | .303 | .05 |

**Table S1b.** Summary of regression analyses using research articles only (N = 432,891) as the unit of analysis in Study 1. Mean Log Cite was the dependent variable. Publication Year was included as a control variable in Model 1 but not in Model 2.

|  | **Model 1** | | | |  | **Model 2** | | | |
| --- | --- | --- | --- | --- | --- | --- | --- | --- | --- |
|  | ***B*** | ***SE*** | ***p*** | ***η_p_^2^*** |  | ***B*** | ***SE*** | ***p*** | ***η_p_^2^*** |
| **Overall data (432891)** |  |  |  |  |  |  |  |  |  |
| Intercept | -.134 | .004 | < .001 | .00 |  | -.103 | .004 | < .001 | .00 |
| Surname Alphabetical Order (A = 1 to Z = 26) | -.014 | .004 | .002 | .00 |  | -.014 | .005 | .002 | .00 |
| System (0 = Alphabetical, 1 = Numerical) | .155 | .004 | < .001 | .00 |  | .119 | .004 | < .001 | .00 |
| Initial Frequency | -.004 | .002 | .026 | .00 |  | -.003 | .002 | .105 | .00 |
| Publication Year | -.195 | .002 | < .001 | .04 |  |  |  |  |  |
| Surname Alphabetical Order x System | .024 | .005 | < .001 | .00 |  | .009 | .005 | .050 | .00 |
|  |  |  |  |  |  |  |  |  |  |
| **Alphabetical System Data** |  |  |  |  |  |  |  |  |  |
| Intercept | .000 | .004 | 1.000 | .00 |  | .000 | .004 | 1.000 | .00 |
| Surname Alphabetical Order (A = 1 to Z = 26) | -.017 | .005 | .001 | .00 |  | -.018 | .005 | .001 | .00 |
| Initial Frequency | -.013 | .005 | .014 | .00 |  | -.013 | .005 | .016 | .00 |
| Publication Year | -.140 | .004 | < .001 | .02 |  |  |  |  |  |
|  |  |  |  |  |  |  |  |  |  |
| **Numerical System Data (372614)** |  |  |  |  |  |  |  |  |  |
| Intercept | .000 | .002 | 1.000 | .00 |  | .000 | .002 | 1.000 | .00 |
| Surname Alphabetical Order (A = 1 to Z = 26) | .013 | .002 | < .001 | .00 |  | -.004 | .002 | .102 | .00 |
| Initial Frequency | -.003 | .002 | .196 | .00 |  | -.001 | .002 | .522 | .00 |
| Publication Year | -.206 | .002 | < .001 | .04 |  |  |  |  |  |

***Employing Poisson Regression Model.*** In the main text, we employed a log-transformed regression model to analyze our data of Study 1. We recognize that this model has limitations, as it does not retain the original scale. However, our primary rationale for selecting log transformation over Poisson regression is that it stabilizes variance and reduces skewness in the data, thereby simplifying the relationship between the independent and dependent variables. Conversely, while Poisson regression does not require transformation and maintains the original scale of counts, it assumes that the mean and variance of those counts are equal. If this assumption is violated, the model may not fit properly, resulting in biased estimates. Furthermore, Poisson regression necessitates positive integers and cannot accommodate datasets that include zero values. Consequently, it may be unsuitable for our data for the following reasons: 1) there are 27,382 cases that include zero in our dataset, accounting for 6.12% of the total cases; and 2) we need to calculate the average value for each surname initial as a unit of analysis, which results in decimal values rather than integers.

We utilized the Poisson regression model solely for the analysis with research articles as the unit of analysis. The results presented in Table S2 demonstrate that the interaction effect between surname alphabetical order and citation system is significant, irrespective of whether publication year is controlled (*ps* ≤ .001). When examining the effects by citation systems, we found that the negative impact of surname order under the alphabetical system is significantly stronger than that under the numerical system, regardless of controlling for publication year (*ps* ≤ .001). In summary, these findings are consistent with those obtained from the log-transformed regression model, reinforcing the validity of our primary conclusions.

**Table S2.** Summary of Poisson regression analyses using articles as the unit of analysis in Study 1. Mean Cite was the dependent variable. Publication Year was included as a control variable in Model 1 but not in Model 2.

|  | **Model 1** | | | |  | **Model 2** | | | |
| --- | --- | --- | --- | --- | --- | --- | --- | --- | --- |
|  | ***B*** | ***SE*** | ***p*** | ***Exp(B)*** |  | ***B*** | ***SE*** | ***p*** | ***Exp(B)*** |
| **Overall data (446755)** |  |  |  |  |  |  |  |  |  |
| Intercept | 34.520 | .028 | < .001 | 9.82E+14 |  | 4.547 | .001 | < .001 | 94.33 |
| Surname Alphabetical Order (A = 1 to Z = 26) | -.009 | 6.49^E-5^ | < .001 | .99 |  | -.009 | 6.477^E-5^ | < .001 | .99 |
| System (0 = Alphabetic, 1 = Numerical) | -.199 | .001 | < .001 | .82 |  | -.212 | .001 | < .001 | .81 |
| Initial Frequency | -7.450^E-8^ | 4.760^E-9^ | < .001 | 1.00 |  | -5.361^E-8^ | 4.748^E-9^ | < .001 | 1.00 |
| Publication Year | -.015 | 1.427^E-5^ | < .001 | .99 |  |  |  |  |  |
| Surname Alphabetical Order x System | .009 | 6.651^E-5^ | < .001 | 1.01 |  | .008 | 6.631^E-5^ | < .001 | 1.01 |
|  |  |  |  |  |  |  |  |  |  |
| **Alphabetical System Data (66734)** |  |  |  |  |  |  |  |  |  |
| Intercept | 48.576 | .067 | < .001 | 1.25E+21 |  | 4.477 | .001 | < .001 | 87.94 |
| Surname Alphabetical Order (A = 1 to Z = 26) | -.006 | 8.044^E-5^ | < .001 | .99 |  | -.006 | 8.031^E-5^ | < .001 | .99 |
| Initial Frequency | 6.218^E-7^ | 1.134^E-8^ | < .001 | 1.00 |  | 6.397^E-7^ | 1.132^E-8^ | < .001 | 1.00 |
| Publication Year | -.022 | 3.346^E-5^ | < .001 | .98 |  |  |  |  |  |
|  |  |  |  |  |  |  |  |  |  |
| **Numerical System Data (380021)** |  |  |  |  |  |  |  |  |  |
| Intercept | 31.176 | .032 | < .001 | 3.46E+13 |  | 4.349 | .001 | < .001 | 77.41 |
| Surname Alphabetical Order (A = 1 to Z = 26) | -3.040^E-4^ | 3.434^E-5^ | < .001 | 1.00 |  | -.002 | 3.419^E-5^ | < .001 | 1.00 |
| Initial Frequency | -2.200^E-7^ | 5.246^E-9^ | < .001 | 1.00 |  | -1.992^E-7^ | 5.234^E-9^ | < .001 | 1.00 |
| Publication Year | -.013 | 1.582^E-5^ | < .001 | .99 |  |  |  |  |  |

***Employing Negative Binomial Regression Model.*** In addition to the Poisson regression model, we also took the negative Binomial regression model into consideration. Similarly, the negative Binomial regression model not only does not require transformation and maintains the original scale of counts; it but also can handle zeros and address overdispersion issue, where variance exceeds the mean. In our data, the mean value is 76.42 while the variance is 63967.2. Thus, we employed the negative Binomial regression model solely for the analysis with research articles as the unit of analysis.

The results presented in Table S3 indicated a significant interaction effect between surname alphabetical order and citation system, irrespective of whether publication year is controlled (*ps* ≤ .001). When divided by citation systems, we found that the negative impact of surname order under the alphabetical system is stronger compared than that under the numerical system, regardless of controlling for publication year (*Bs* ≤ -.0003). In sum, these findings are consistent with those obtained from the log-transformed regression model, enhancing the robustness of our primary conclusions.

***Controlling for Journal Outlet.*** Furthermore, from an exploratory perspective, we examine if journal outlet will influence our current findings. Accordingly, we employed a negative binomial regression model, incorporating journal outlets as a level 2 controlled variable. As shown in Table S4, we found that the results after controlling for journal outlets were consistent with those obtained without this control. Specifically, after controlling for journal outlets, the interaction between surname alphabetical order and citation system was highly significant across the overall dataset (*ps* ≤ .001), regardless of whether publication year was controlled. When analyzing the data by citation systems, with controlling for publication year, the influence of surname alphabetical order under the alphabetical system was negative (*B* = -.003, *p* = .003), whereas the effect under the numerical system was positive (*B* = .002, *p* < .001). Without controlling for publication year, the influence of surname alphabetical order under the alphabetical citation system remained negative (*B* = -.003, *p* = .005), while the positive effect of surname alphabetical order under the numerical citation system became non-significant (*p* = .925). All together, our main conclusion remains robust, irrespective of whether journal outlets are controlled.

***Controlling for Publication Year as a Random Effect.*** Additionally, we consider the publication years may be a random effect rather than a linear fixed effect, such as the effect of publication years on total citations may differ across different contexts (e.g., fields and topics). Accordingly, we employed a negative binomial regression model, incorporating publication years as a level 2 controlled variable. As shown in Table S5, the interaction between surname alphabetical order and citation system was highly significant across the overall dataset (*p* < .001). When analyzing the data by citation systems, the influence of surname alphabetical order under the alphabetical system was negative (*B* = -.004, *p* < .001), whereas the effect under the numerical system was positive (*B* = .001, *p* < .001). In sum, the main conclusion remains robust, irrespective of how publication years are controlled.

***Controlling for Author’s Nationality.*** Lastly, we notice that incorporating author-country fixed effects into the model can help mitigate potential confounders. Thus, we employed the name-ethnicity classification (NEC) algorithm developed by Hafner, Peifer, and Hafner (2024). Compared to other available algorithms (e.g., NamSor, NamePrism, EthnicityEstimator, and Ethnicolr), the NEC algorithm provides a more balanced dataset across ethnicity, gender, and age. It is also more efficient in processing speed and offers enhanced accuracy and inclusivity, all while being freely accessible. Specifically, the NEC algorithm infers nationality based on first and last names and provides a corresponding confidence rate (see <https://github.com/name-ethnicity-classifier/name-ethnicity-classifier>). Among the various models offered, we selected the "21_nationalities_and_else" model, as it achieves an accuracy rate of 81.08% while encompassing a sufficient range of nationalities. Accordingly, we employed a negative binomial regression model while controlling for nationality as a random effect factor to let the intercept vary across nationalities. We did not control nationality as a fixed effect factor as we were not interested in estimating specific nationality effects.. Table S6 presents the number of first authors corresponding to each country in the entire sample. Table S7 reports the analysis results. Specifically, the interaction between surname alphabetical order and citation system was significant across the overall dataset (*ps* < .001). After controlling for nationality, our results appeared more robust, as all the influences of surname alphabetical order were consistently negative (*Bs* ≤ -.006) regardless of the citation system. Notably, these negative effects were only significant under the alphabetical system, even when simultaneously controlling for publication year (*ps* ≤ .001). Overall, our main conclusion remains robust, irrespective of nationality control.

**Discussion**

Importantly, there is notable discrepancies between the results of the Poisson and negative binomial models and those derived from linear models with logged outcomes. For example, the negative binomial model indicated a beta coefficient of -.006 for the surname alphabetical order, translating to a modest expected citation rate decrease of 0.6% per unit increase. In contrast, the linear models suggested a more substantial reduction of 41% between authors with surnames from A to Z. These differences highlight the importance of model selection in interpreting our findings as well as the assumptions regarding the data nature, distributions, and variances. The negative binomial model, which accounts for overdispersion in count data, provides a different perspective on the influence of surname alphabetical order and interpretations of coefficients (i.e. whether it is a direct change, or multiplicative effect on the expected count). Overall speaking, the results in different models showing a similar pattern of findings affirm that our main conclusions in this study remain valid. We also suggest that future research could further explore the implications of these model choices.

**Table S3.** Summary of negative Binomial regression analyses using articles as the unit of analysis in Study 1. Mean Cite was the dependent variable. Publication Year was included as a control variable in Model 1 but not in Model 2.

|  | **Model 1** | | | |  | **Model 2** | | | |
| --- | --- | --- | --- | --- | --- | --- | --- | --- | --- |
|  | ***B*** | ***SE*** | ***p*** | ***95% CI*** |  | ***B*** | ***SE*** | ***p*** | ***95% CI*** |
| **Overall data (446755)** |  |  |  |  |  |  |  |  |  |
| Intercept | 49.365 | .321 | < .001 | [48.736, 49.993] |  | 4.546 | .012 | < .001 | [4.529, 4.562] |
| Surname Alphabetical Order (A = 1 to Z = 26) | -.008 | .001 | < .001 | [-.009, -.007] |  | -.009 | .001 | < .001 | [-.010, -.008] |
| System (0 = Alphabetic, 1 = Numerical) | -.139 | .008 | < .001 | [-.155, -.123] |  | -.209 | .011 | < .001 | [-.224, -.193] |
| Initial Frequency | -9.426^E-8^ | 4.186^E-8^ | .024 | [.000, .000] |  | -7.318^E-8^ | 4.194^E-8^ | .206 | [-1.554^E+7^, 9.011^E-9^] |
| Publication Year | -.022 | 2.000^E-4^ | < .001 | [-.023, -.022] |  |  |  |  |  |
| Surname Alphabetical Order x System | .009 | .001 | < .001 | [.007, .010] |  | .007 | .001 | < .001 | [.006, .008] |
|  |  |  |  |  |  |  |  |  |  |
| **Alphabetic System Data (66734)** |  |  |  |  |  |  |  |  |  |
| Intercept | 79.705 | .836 | < .001 | [78.066, 81.343] |  | 4.477 | .021 | < .001 | [4.452, 4.503] |
| Surname Alphabetical Order (A = 1 to Z = 26) | -.006 | .001 | < .001 | [-.007, -.004] |  | -.006 | .001 | < .001 | [-.007, -.004] |
| Initial Frequency | 3.22^E-07^ | 1.06^E-07^ | .060 | [1.137^E-7^, 5.300^E-7^] |  | 6.121^E-7^ | 1.066^E-7^ | < .001 | [4.032^E-7^, 8.209^E-7^] |
| Publication Year | -.038 | 4.866^E-4^ | < .001 | [-.038, -.037] |  |  |  |  |  |
|  |  |  |  |  |  |  |  |  |  |
| **Numerical System Data (380021)** |  |  |  |  |  |  |  |  |  |
| Intercept | 43.803 | .3469 | < .001 | [43.123, 44.483] |  | 4.349 | .007 | < .001 | [4.339, 4.360] |
| Surname Alphabetical Order (A = 1 to Z = 26) | -.0003 | 3.974^E-4^ | .544 | [-.001, .000] |  | -.002 | 3.997^E-4^ | < .001 | [-.003, -.001] |
| Initial Frequency | -1.929^E-07^ | 4.554^E-08^ | .002 | [-2.821^E-7^, -1.036^E-7^] |  | -2.010^E-7^ | 4.561^E-8^ | .001 | [-2.904^E-7^, -1.116^E-7^] |
| Publication Year | -.020 | 1.889^E-4^ | < .001 | [-.020, -.019] |  |  |  |  |  |
| *Note. 95% Wald Confidence Interval.* |  |  |  |  |  |  |  |  |  |

**Table S4.** Summary of negative Binomial regression analyses with Journal Outs as level 2 controlled variable, and using articles as the unit of analysis in Study 1. Mean Cite was the dependent variable. Publication Year was included as a control variable in Model 1 but not in Model 2.

|  | **Model 1** | | |  | **Model 2** | | |
| --- | --- | --- | --- | --- | --- | --- | --- |
|  | ***B*** | ***SE*** | ***p*** |  | ***B*** | ***SE*** | ***p*** |
| **Overall data (446755)** |  |  |  |  |  |  |  |
| Intercept | 43.698 | .439 | < .001 |  | 4.236 | .161 | < .001 |
| Surname Alphabetical Order (A = 1 to Z = 26) | -.005 | 7.289^E-4^ | < .001 |  | -.005 | 7.359^E-4^ | < .001 |
| System (0 = Alphabetic, 1 = Numerical) | .225 | .238 | .345 |  | .103 | .245 | .674 |
| Initial Frequency | 1.148^E-7^ | 4.844^E-8^ | .018 |  | 1.440^E-7^ | 4.870^E-8^ | .003 |
| Publication Year | -.020 | 2.056^E-4^ | < .001 |  |  |  |  |
| Surname Alphabetical Order x System | .007 | 7.477^E-4^ | < .001 |  | .005 | 7.549^E-4^ | < .001 |
|  |  |  |  |  |  |  |  |
| **Alphabetic System Data (66734)** |  |  |  |  |  |  |  |
| Intercept | 72.130 | 1.293 | < .001 |  | 4.194 | .162 | < .001 |
| Surname Alphabetical Order (A = 1 to Z = 26) | -.003 | .001 | .003 |  | -.003 | .002 | .005 |
| Initial Frequency | 3.999^E-7^ | 1.480^E-7^ | .007 |  | 5.642^E-7^ | 1.512^E-7^ | < .001 |
| Publication Year | -.034 | 6.427^E-4^ | < .001 |  |  |  |  |
|  |  |  |  |  |  |  |  |
| **Numerical System Data (380021)** |  |  |  |  |  |  |  |
| Intercept | 39.041 | 0.466 | < .001 |  | 4.346 | .184 | < .001 |
| Surname Alphabetical Order (A = 1 to Z = 26) | .002 | 3.521^E-4^ | < .001 |  | 3.347^E-5^ | 3.549^E-4^ | .925 |
| Initial Frequency | 5.278^E-8^ | 5.077^E-8^ | .030 |  | 6.753^E-8^ | 5.123^E-8^ | .187 |
| Publication Year | -.017 | 2.142^E-4^ | < .001 |  |  |  |  |

**Table S5.** Summary of negative Binomial regression analyses with Publication Years as level 2 controlled variable, and using articles as the unit of analysis in Study 1. Mean Cite was the dependent variable.

|  | ***B*** | ***SE*** | ***p*** |
| --- | --- | --- | --- |
| **Overall data (446,755)** |  |  |  |
| Intercept | 4.351 | .102 | < .001 |
| Surname Alphabetical Order (1 = “A” to 26 = “Z”) | -.008 | .001 | < .001 |
| System (0 = Alphabetic, 1 = Numerical) | -.132 | .010 | < .001 |
| Initial Frequency | -.784^E-07^ | .502^E-07^ | .118 |
| Surname Alphabetical Order x System | .010 | .001 | < .001 |
|  |  |  |  |
| **Alphabetic System Data (66,734)** |  |  |  |
| Intercept | 4.264 | .153 | < .001 |
| Surname Alphabetical Order (1 = “A” to 26 = “Z”) | -.004 | .001 | < .001 |
| Initial Frequency | .441^E-06^ | .144^E-06^ | .002 |
|  |  |  |  |
| **Numerical System Data (380,021)** |  |  |  |
| Intercept | 4.224 | .097 | < .001 |
| Surname Alphabetical Order (1 = “A” to 26 = “Z”) | .001 | .367^E-03^ | .013 |
| Initial Frequency | -.217^E-06^ | .531^E-07^ | < .001 |

**Table S6.** Frequency of each nationality in the sample for Study 1.

| **Nationality** | **N** | **%** |
| --- | --- | --- |
| British | 44371 | 9.9% |
| Bulgarian | 1758 | 0.4% |
| Chinese | 59250 | 13.3% |
| Danish | 34425 | 7.7% |
| Dutch | 26788 | 6.0% |
| French | 25672 | 5.7% |
| German | 44888 | 10.0% |
| Greek | 4914 | 1.1% |
| Hungarian | 7547 | 1.7% |
| Indian | 12961 | 2.9% |
| Italian | 20186 | 4.5% |
| Japanese | 19625 | 4.4% |
| Nigerian | 4072 | 0.9% |
| Pakistani | 4700 | 1.1% |
| Polish | 11368 | 2.5% |
| Romanian | 4198 | 0.9% |
| Russian | 8032 | 1.8% |
| Spanish | 8370 | 1.9% |
| Swedish | 15599 | 3.5% |
| Turkish | 3937 | 0.9% |
| Ukrainian | 4373 | 1.0% |
| Zimbabwean | 4949 | 1.1% |
| *Else* | 74772 | 16.7% |
| **Total** | **446755** | **100%** |
|  | | |

**Table S7.** Summary of negative Binomial regression analyses while controlling for first author’s Nationality as a random effect factor, and using articles as the unit of analysis in Study 1. Mean Cite was the dependent variable. Publication Year was included as a control variable in Model 1 but not in Model 2.

|  | | | | | | | |
| --- | --- | --- | --- | --- | --- | --- | --- |
|  | **Model 1** | | |  | **Model 2** | | |
|  | ***B*** | ***SE*** | ***p*** |  | ***B*** | ***SE*** | ***p*** |
| **Overall data (446755)** |  |  |  |  |  |  |  |
| Intercept | 46.181 | .463 | < .001 |  | 4.468 | .031 | < .001 |
| Surname Alphabetical Order (1 = “A” to 26 = “Z”) | -.008 | .001 | < .001 |  | -.008 | .001 | < .001 |
| System (0 = Alphabetic, 1 = Numerical) | -.126 | .011 | < .001 |  | -.175 | .011 | < .001 |
| Initial Frequency | -.880^E-07^ | 5.422^E-08^ | .105 |  | -.903^E-07^ | .545^E-07^ | .098 |
| Publication Year | -.021 | 2.315^E-04^ | < .001 |  |  |  |  |
| Surname Alphabetical Order x System | .008 | .001 | < .001 |  | .008 | .001 | < .001 |
|  |  |  |  |  |  |  |  |
| **Alphabetic System Data (66734)** |  |  |  |  |  |  |  |
| Intercept | 76.179 | 1.331 | < .001 |  | 4.399 | .054 | < .001 |
| Surname Alphabetical Order (1 = “A” to 26 = “Z”) | -.006 | .001 | < .001 |  | -.006 | .001 | < .001 |
| Initial Frequency | .241^E-06^ | 1.574^E-07^ | .125 |  | .425^E-06^ | .161^E-06^ | .008 |
| Publication Year | -.036 | .001 | < .001 |  |  |  |  |
|  |  |  |  |  |  |  |  |
| **Numerical System Data (380021)** |  |  |  |  |  |  |  |
| Intercept | 40.405 | .491 | < .001 |  | 4.300 | .029 | < .001 |
| Surname Alphabetical Order (1 = “A” to 26 = “Z”) | -.365^E-04^ | .401^E-03^ | .927 |  | -.600^E-03^ | .402^E-03^ | .137 |
| Initial Frequency | -.175^E-06^ | .575^E-07^ | .002 |  | -.195^E-06^ | .578^E-07^ | .001 |
| Publication Year | -.018 | .245^E-03^ | < .001 |  |  |  |  |

**Figure S1.** Visualization of the Experimental Design of Study 2. It should be noted that this figure presents a simplified version of the experimental design and does not include the conditions related to the article versions.

**
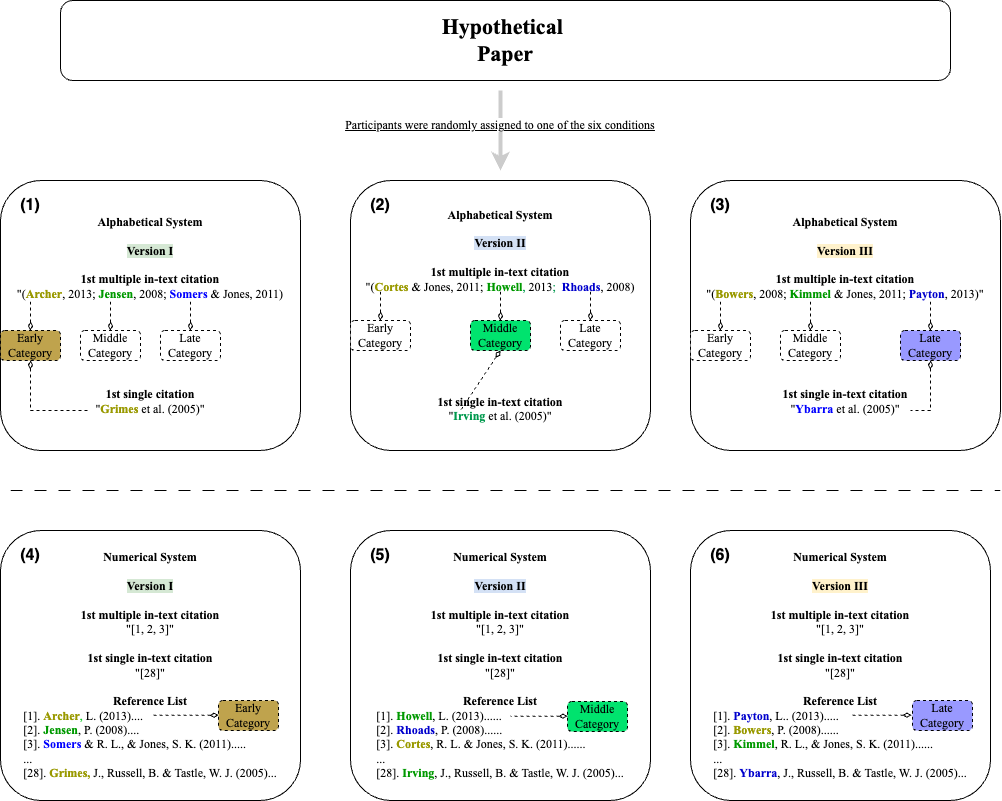
**

**Figure S2.** Mean Citation Frequency of Each Reference Order Category by Citation Systems and Article Versions in Study 2.


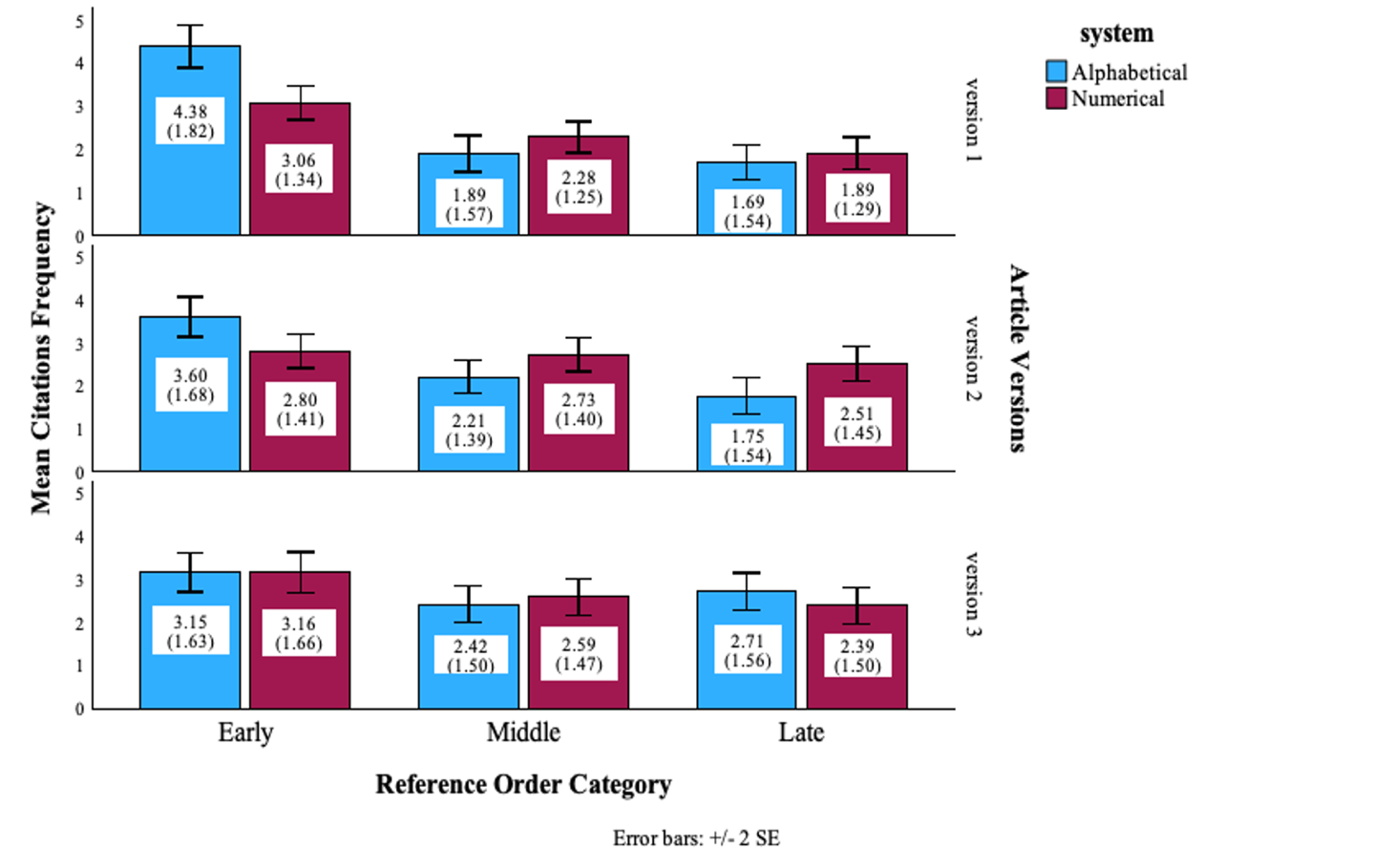


**Reference**

Hafner, L., Peifer, T. P., & Hafner, F. S. (2024). Equal accuracy for Andrew and Abubakar—detecting and mitigating bias in name-ethnicity classification algorithms. *AI & Society, 39*(4), 1605-1629.
